# Preparation of safflower fermentation solution and study on its biological activity

**DOI:** 10.3389/fmicb.2024.1472992

**Published:** 2024-10-30

**Authors:** Nan Tang, Xiaoqing Xu, Zhenyu Guo, Xiangyu Meng, Guoqiang Qian, He Li

**Affiliations:** ^1^School of Base Medical Sciences, Guangdong Pharmaceutical University, Guangzhou, China; ^2^School of Chinese Medicine, Guangdong Pharmaceutical University, Guangzhou, China

**Keywords:** safflower, yeast, fermentation, antioxidant, anti-inflammatory

## Abstract

**Introduction:**

Safflower, a traditional Chinese medicine, is rich in chemical components including flavonoids, polysaccharides, and alkaloids. It exhibits pharmacological effects such as antioxidant, anti-inflammatory, anti-tumor, and anti-thrombosis properties, making it a valuable resource in the medical field. Furthermore, due to its antioxidant and anti-inflammatory effects, safflower is increasingly being utilized in the cosmetics industry.

**Methods:**

In this study, yeast was employed to ferment safflower, and the optimal fermentation conditions were established through single-factor experiments and response surface methodology. Subsequently, the antioxidant and anti-inflammatory efficacy of the safflower fermentation solution was assessed using both cellular and zebrafish models. Finally, the safety of the safflower fermentation solution was evaluated through a cosmetic eye irritation test.

**Results:**

From a total of 20 yeast strains, YF-5 was identified as the dominant strain for safflower fermentation. By optimizing the fermentation conditions, it was established that the optimal parameters for YF-5 fermentation of safflower are as follows: a fermentation temperature of 36.55°C, a material-to-liquid ratio of 1:20.46, a fructose concentration of 6.20%, a fermentation duration of 72 h, and an inoculum volume of 4%. The biological activities of safflower, including its antioxidant and anti-inflammatory properties, were enhanced through yeast fermentation. In HaCaT cell and zebrafish oxidative damage assays, safflower fermentation solution inhibits the production of malondialdehyde (MDA) and increases superoxide dismutase (SOD) activity as well as total antioxidant capacity (T-AOC). In the RAW264.7 cell inflammatory damage assays, a 20% safflower fermentation solution was found to inhibit the release of TNF-α and NO in the inflammatory model, with inhibition rates of 30.94 and 28.86%, respectively. In the zebrafish inflammatory damage assays, the quantity of fluorescent neutral proteins in the 5% safflower fermentation solution was 0.7 times that observed in the dexamethasone (0.1 mg/mL) positive control group, indicating that its anti-inflammatory activity is comparable to that of dexamethasone (0.1 mg/mL). In the chicken embryo chorionic membrane experiment, it was observed that the safflower fermentation solution did not cause significant damage to the blood vessels of the chorionic allantoic membrane (CAM). This finding demonstrates that the safflower fermentation solution possesses a certain degree of safety.

**Discussion:**

Safflower fermentation solution has antioxidant and anti-inflammatory bioactivities, and it has passed cosmetic safety evaluations. It can be used as a new natural cosmetic ingredient added to cosmetic products.

## 1 Introduction

Safflower (*Carthamus tinctorius* L.) is a member of the Asteraceae family and its dried flowers are used as a traditional Chinese medicinal herb and food plant in China (Lin et al., 2021). In China, safflower is mainly distributed in Henan, Xinjiang, Inner Mongolia and Yunnan (Ying et al., 2015). The material components in safflower primarily consist of flavonoids, polysaccharides, alkaloids, and other compounds. Among these, flavonoids and polysaccharides are the main active components of safflower, exhibiting antioxidant (Zemour et al., 2019; Ozkan et al., 2021; Temirbekova et al., 2019; Jung et al., 2019), anti-inflammatory (Rusli et al., 2023; Chen et al., 2023), anti-tumour (Fan, 2013; Meng et al., 2018), anti-thrombotic (Fan et al., 2009; Kuehnl et al., 2013; Yang et al., 2015) and other pharmacological activities. The active components and pharmacological effects of safflower are research hotspots for scholars both domestically and internationally. Safflower is frequently utilized in the medical field for the treatment of various conditions, including gynecological, cardiovascular, and cerebrovascular diseases. It demonstrates promising applications in improving myocardial ischemia, coagulation, thrombosis, and inflammation (Delshad et al., 2018). Additionally, safflower has the potential to prevent skin photoaging and may be used in the formulation of anti-wrinkle and skin health-enhancing cosmetics, indicating its potential applications in the cosmetics industry (Jeong et al., 2020).

Plants contain a variety of active substances, including polysaccharides, polypeptides, flavonoids, polyphenols, and amino acids. However, the concentration of these active ingredients in natural plant raw materials is generally low. Fermenting plants using microorganisms can significantly increase the levels of active ingredients and enhance their biological activity. The extraction of bioactive substances from plant fermentation primarily relies on the co-fermentation of microorganisms and plants. Through the interaction between microorganisms and plants that have both medicinal and nutritional properties, the efficacy of the active compounds in these plants is improved, thereby enhancing their biological activities (Xia et al., 2024). Gadhoumi et al. (2021) discovered that the fermentation of *Lactobacillus acidophilus* with *ficus-indica* and *Lavandula multifida* resulted in the transformation and enrichment of these plants with active ingredients. This process of microbial and botanical co-fermentation enhanced their antibacterial and antifungal properties. Wei et al. (2022) conducted a study where selenium-enriched mung bean, *Lactobacillus helveticus*, and Bifidobacteria were co-fermented at 37°C for 50 h in a fermenter to produce a mung bean fermentation solution. This solution was assessed for its efficacy and showed notable anti-aging, whitening, and moisturizing properties. The co-fermentation of microorganisms and plants facilitates the extraction and transformation of active ingredients in plants, leading to higher content of these compounds, improved biological activity, and the development of novel cosmetic raw materials for the beauty industry.

The aim of this study was to prepare a safflower fermentation solution through co-fermentation of microorganisms with safflower, and to explore its potential application as a novel cosmetic raw material. The study involved screening yeast strains suitable for safflower fermentation from a pool of 20 strains in the laboratory. Subsequently, the fermentation conditions for safflower yeast fermentation were optimized through single factor tests and response surface methodology. The antioxidant and anti-inflammatory effects of the safflower fermentation solution were then examined using cellular and zebrafish models. Finally, the safety of the safflower fermentation solution was assessed through cosmetic eye irritation experiments. This study investigates the use of microorganisms to ferment safflower, enhancing its biological activity. The objective is to develop a safflower fermentation solution that exhibits antioxidant and anti-inflammatory properties, thereby creating a novel cosmetic raw material for application in the cosmetics industry.

## 2 Materials and methods

### 2.1 Materials and reagents

Safflower was purchased from Baichuntang Pharmaceuticals, the yeasts used in the study were kept by the Laboratory of Biochemistry and Molecular Biology, Guangdong Pharmaceutical University. Potato dextrose medium (PDB), 1,640 medium, DMEM medium, trypsin, and bacterial lipopolysaccharide (LPS) were sourced from Thermo Fisher Scientific. Total antioxidant capacity (T-AOC) kit, malondialdehyde (MDA) kit, total superoxide dismutation (T-SOD) kit, hydrogen peroxide (CAT) kit, Caulmers Brilliant Blue (CMB) kit, and NO kit were acquired from Nanjing Jianjian Bio-Technology Co. Mouse mononuclear macrophages (RAW264.7) were obtained from Delph Biotics. Fluorescent neutrophil-containing zebrafish Tg (corola: EGFP) was sourced from the zebrafish laboratory supply of Guangdong Pharmaceutical University. Human keratinocytes (HaCaT) were purchased from Wonders Biological Co. AB line zebrafish embryos were obtained from Shanghai Fei Xi Biotechnology Co.

### 2.2 Optimization of safflower fermentation process

#### 2.2.1 Activation of safflower fermentation strains

Twenty yeast species were retrieved from an ultra-low temperature refrigerator, then thawed at 37°C and inoculated in PDB liquid medium. The cultures were incubated at 37°C with a shaking rate of 220 rpm for 6 h. The absorbance value of the yeast solution at 630 nm was measured using an enzyme marker, and efforts were made to maintain the absorbance value within the range of 0.8–1.2.

#### 2.2.2 Preparation of safflower fermentation solution

Weigh 1 g of safflower powder into a 100 mL conical bottle, then add 20 mL of sterile water following a 1:20 (g/v) ratio of material to liquid. Next, add 0.4 mL of a different bacterial liquid to the safflower water at a 2% (v/v) ratio of bacterial liquid addition. Repeat this process for a total of 20 sample groups. Sterile water was added to the safflower feed water instead of a bacterial solution to serve as a control group for fermentation. The 21 experimental groups were then placed in a constant temperature shaker set at 37°C and a rate of 220 rpm for a 48-h fermentation period. Following completion of fermentation, the safflower fermentation solutions from each group were aliquoted into 5 mL centrifuge tubes. The tubes were then centrifuged at 4°C and 9,000 rpm for 5 min, after which the supernatant was collected and filtered through a 0.22 μm filter membrane to eliminate bacteria. The resulting safflower fermentation solutions were used for subsequent experiments.

#### 2.2.3 Screening of safflower fermentation strains

Polysaccharides, flavonoids, and antioxidant capacity were used as evaluation indexes to select the most suitable yeast for safflower fermentation. Polysaccharides, flavonoids, and antioxidant capacity were used as evaluation indexes to select the most suitable yeast for safflower fermentation.

The total polysaccharide content in plants is commonly determined using the phenol sulphate method (Shu-ya, 2014). In a 10 mL centrifuge tube, sequentially add 1 mL of sample, 1 mL of 5% phenol solution, and 5 mL of concentrated sulphuric acid. Allow the mixture to rest for 10 min, then shake the reaction system well. Proceed to react for 30 min at room temperature, followed by measuring the absorbance values of each group at 490 nm after the reaction is complete. The vertical coordinate represented the glucose content while the horizontal coordinate represented the absorbance value. Subsequently, a standard curve was plotted.

The total flavonoid content of the safflower fermentation solution was determined using the aluminium nitrate chromogenic method (Bing-sheng, 2012). The method involved adding 1 mL of the sample to a 10 mL centrifuge tube, followed by the addition of 1 mL of 5% sodium nitrite solution. After shaking and allowing the reaction to rest for 6 min, 1 mL of 10% aluminium nitrate solution was added, followed by another round of shaking and resting for 6 min. Subsequently, 4 mL of 4% sodium hydroxide solution was added, and the mixture was topped up with double-distilled water to reach the 10 mL mark. The reaction was then left to proceed for 15 min. Upon completion of the reaction, the sample’s absorbance was measured at 510 nm. A standard curve was then constructed with the rutin content represented on the vertical axis and the absorbance value on the horizontal axis.

The antioxidant capacity of safflower fermentation solution was assessed using the salicylic acid method (Abduxukur et al., 2023). The experimental procedure involved setting up the reaction system as detailed in Table 1, shaking it well, and allowing it to react statically in a constant temperature water bath at 37°C for 30 min. Following the reaction, the absorbance values of each group were measured at 510 nm, and the hydroxyl radical scavenging capacity of the safflower fermentation solutions in each group was calculated using Equation 1.


(1) $$\mathrm{Hydroxyl}\;\mathrm{radical}\;\mathrm{clearance}\;\left(\%\right)=\left[1-\left(\frac{A_{X}-A_{1}}{A_{0}}\right)\right]*100\%$$

**Table 1:** Salicylic acid reaction system.

Reagents	Volume of solution added to the reaction system/mL		
	*A*_0_	*A*_X_	*A*_1_
FeSO_4_	1	1	1
Salicylic acid	1	1	0
Anhydrous ethanol	1	0	1
Sample	0	1	1
H_2_O_2_	1	1	1

*A0 represents the absorbance value of the normal reaction group, AX represents the absorbance value of the reaction group with the addition of safflower fermentation solution, and A1 represents the absorbance value of the solution blank control group.*

#### 2.2.4 Optimisation of safflower fermentation process system by response surface methodology

##### 2.2.4.1 Single-factor experiment

The single factor method was employed to study the fermentation process conditions of safflower. The total flavonoids of safflower were used as an indicator to examine the impact of six fermentation conditions: carbon source type, carbon source concentration, bacterial liquid inoculation amount, solid–liquid ratio, fermentation temperature, and fermentation time on YF-5 fermentation of safflower.

##### 2.2.4.2 Plackett–Burman design

The P–B experiment focused on examining the impact of different factors on the total flavonoid content of safflower fermentation solution. The variables selected as the low and high levels were based on the total flavonoid content of safflower before and after reaching its maximum value in each fermentation condition. The experimental design included varying levels of material-liquid ratio (1 g:15 mL at low level and 1 g:25 mL at high level), bacterial broth inoculum (2% at low level and 8% at high level), fructose concentration (2% at low level and 8% at high level), fermentation duration (48 h at low level and 96 h at high level), and fermentation temperature (34°C at low level and 41°C at high level). These factors were inputted into Design-Expert 10 software for analysis, resulting in the creation of 12 experimental protocols based on the total flavonoid content in safflower fermentation solution as a key indicator, as outlined in Table 2.

**Table 2:** Plackett–Burman experimental design and results.

Run	A	B	C	D	E	Total flavonoid content/mg
1	25	2	8	96	34	66.24
2	25	2	8	96	41	85.47
3	15	8	2	96	41	27.82
4	15	2	2	96	34	25.38
5	25	2	2	48	41	40.13
6	15	8	8	96	34	69.99
7	15	2	2	48	34	28.50
8	15	2	8	48	41	70.88
9	25	8	2	96	41	47.88
10	15	8	8	48	41	63.59
11	25	8	2	48	34	30.58
12	25	8	8	48	34	61.12

##### 2.2.4.3 Box–Behnken design

Three main factors affecting safflower fermentation were determined through PB experimental design. A total of 17 experimental groups were designed to analyze the flavonoid content in safflower fermentation solution. The pre-maximum, maximum, and post-maximum conditions of three factors were substituted into Design-Expert 10 software to create these experimental designs.

### 2.3 Investigation of *in vitro* antioxidant activity of safflower fermentation solution

HaCaT cells stored at −80°C were thawed and resuscitated to obtain a cell suspension. The cells were then inoculated into T25 cell culture flasks containing 3 mL of fully cultivated DMEM medium, and the cell culture was conducted at 37°C with 5% CO_2_. Upon reaching 80 to 90% confluency, the cells were observed, and the HaCaT cell suspension in good condition was aspirated. A 0.4% Trypan blue solution was added in a 1:1 ratio, and the mixture was dripped onto the cell counting plate. The cell density was adjusted to 1 × 10^5^ cells/mL, and the HaCaT cell suspension was prepared accordingly. Safflower fermentation solution was diluted with basal DMEM medium at concentrations of 40, 30, 20, 10, 5, 2.5, 1.25, and 0.625%.

#### 2.3.1 Evaluation of safe concentration of safflower fermentation solution on HaCaT cells

Sterile PBS solution was added to the perimeter of a 96-well cell culture plate. The remaining wells were then divided into experimental, blank, and normal groups. Each well, except for those in the blank group, received 100 μL of cell suspension. Cell culture was conducted at 37°C with 5% CO_2_. The experimental group received 100 μL of safflower fermentation solution at varying concentrations (40, 30, 20, 10, 5, 2.5, 1.25, 0.625%), while the normal group received 100 μL of basal DMEM medium. The cells were incubated in a constant temperature incubator at 37°C with 5% CO_2_ for 20 h. Subsequently, 50 μL of MTT solution was added to each well, followed by a 4-h incubation period. Afterward, 150 μL of DMSO solution was added and the absorbance values were measured using an enzyme marker at a wavelength of 570 nm. The cell survival rate of HaCaT was determined using Equation 2. Safflower fermentation solutions with a cell survival rate of 50% or higher were chosen for further experiments at varying concentrations of high, medium, and low.


(2) $$\mathrm{Cell}\;\mathrm{survival}\;\mathrm{rate}\;\left(\%\right)={\frac{\left(A_{X}-A_{0}\right)}{\left(A_{1}-A_{0}\right)}}^{*}\;100\%$$

where letter *A*_X_: absorbance value of safflower experimental group; *A*_0_: absorbance value of solvent blank group; *A*_1_: absorbance value of normal group.

#### 2.3.2 Preparation of antioxidant cell samples from safflower fermentation solution

Hydrogen peroxide (H_2_O_2_) is known for its high oxidizing properties which can lead to the oxidation of intracellular reduced glutathione, resulting in the production of hydroxyl radicals (OH^−^) that can cause damage to cells. In this study, H_2_O_2_ was selected to create an oxidative model for the cells, while vitamin C (Vc), a well-known antioxidant, was used as a positive control.

The normal group received 10 μL of basal DMEM medium, while the remaining groups received 10 μL of hydrogen peroxide solution to achieve a final concentration of 0.25 mM in each well. These groups were categorized as the model, positive, high concentration, medium concentration, and low concentration groups, and were exposed to oxidative stimulation for 12 h at 37°C and 5% CO_2_. Cell samples were collected and labelled from each well at the end of the modelling process. The protein concentration of each cell sample group was determined using the Coomassie Brilliant Blue method, which was then utilized to assess the antioxidant activity of the safflower fermentation solution.

### 2.4 Investigation of *in vivo* antioxidant activity of safflower fermentation solution

Zebrafish embryos at the embryonic shield stage were selected and 15 embryos were placed in each well of a 6-well plate. The first well received 3 mL of embryo culture medium, while the second to sixth wells were treated with 3 mL of safflower fermentation solution diluted with the embryo culture medium to concentrations of 10, 5, 2.5, 1.25, and 0.625%, respectively. Zebrafish embryos were exposed to appropriate lighting conditions in a 28°C incubator with a 14:10 ratio of light to darkness. The culture medium was refreshed in the morning and evening for each experimental group. The mortality rate, hatching rate and deformity rate of zebrafish embryos were observed and recorded under a stereomicroscope at three time points: 24 h post-fertilization (hpf), 48 hpf, and 72 hpf. The cumulative results were analyzed using a specific Equations 3–5 to determine the concentration of safflower fermentation solution suitable for subsequent zebrafish embryo experiments in three parallel trials.


(3) $$\mathrm{Mortality}\;\mathrm{rate}\;\left(\%\right)=100-\left(\frac{\mathrm{Number}\;\mathrm{of}\;\mathrm{dead}\;\mathrm{embryos}}{\mathrm{Total}\;\mathrm{number}\;\mathrm{of}\;\mathrm{embryos}}\times 100\right)$$

(4) $$\mathrm{Hatching}\;\mathrm{rate}\;\left(\%\right)=\left(\frac{\mathrm{Number}\;\mathrm{of}\;\mathrm{embryos}\;\mathrm{hatched}}{\mathrm{Total}\;\mathrm{number}\;\mathrm{of}\;\mathrm{embryos}}\right)\times 100\%$$

(5) $$\mathrm{Deformity}\;\mathrm{rate}\;\left(\%\right)=\left(\frac{\mathrm{Number}\;\mathrm{of}\;\mathrm{deformed}\;\mathrm{embryos}}{\mathrm{Total}\;\mathrm{number}\;\mathrm{of}\;\mathrm{embryos}}\right)\times 100\%$$

#### 2.4.1 Preparation of antioxidant samples from AB line zebrafish embryos

Zebrafish embryos at the embryonic shield stage were individually selected and 15 embryos were placed into each well of a 6-well plate. The first and second wells received 3 mL of standard embryo culture solution, designated as the normal and model groups, respectively. The third well was treated with 3 mL of vitamin C solution at a concentration of 10 μg/mL, serving as the positive control group. The fourth to sixth wells were each treated with 3 mL of safflower fermentation solution at varying concentrations within the safe range, from high to medium-low. Subsequently, the plate was incubated in a 28°C incubator. Following 72 h post-fertilization (hpf) treatment, 3 mL of embryo culture solution was added to the normal group, while the remaining groups received 3 mL of AAPH solution at a concentration of 1 mM to induce oxidative stress. Incubation was then continued for 6 h in an incubator, after which zebrafish were collected from each well. Zebrafish tissue homogenate was prepared using saline as the solvent, followed by centrifugation at 10,000 rpm for 10 min at 4°C. The resulting supernatant was used as the zebrafish samples for testing in each well, and the protein concentration of each sample group was determined using the Caumas Brilliant Blue method.

### 2.5 Determination of total antioxidant capacity, malondialdehyde content and superoxide dismutase activity

#### 2.5.1 Determination of total antioxidant capacity

In accordance with the guidelines outlined in the total antioxidant capacity kit using a colourimetric method, samples from HaCaT (2.3.2) and zebrafish (2.4.2) were collected and the absorbance values of each sample were determined. These values were then utilized in Equation 6 to calculate the total antioxidant capacity for each group, which was expressed in U/mL.


(6) $$\begin{array}{l} \mathrm{Total}\;\mathrm{antioxidant}\;\mathrm{capacity}\\ =\frac{A_{\mathrm{meauring}\;\mathrm{tube}}-A_{\mathrm{control}\;\mathrm{tube}}}{0.01}÷30\times 3.7 \end{array}$$

#### 2.5.2 Determination of malondialdehyde content

Following the guidelines outlined in the malondialdehyde kit using the TBA method, a variety of reagents were combined. The reaction took place in a water bath at 95°C for 40 min, followed by cooling and centrifugation at 4,500 rpm for 10 min. The supernatant was then collected and the absorbance value of each sample was measured at 532 nm. The MDA content in each sample group was determined by substituting values into Equation 7.


(7) $$MDA\;\mathrm{content}=\frac{\mathrm{Measured}\;OD\;\mathrm{value}-OD\;\mathrm{values of control}}{\begin{array}{l} \mathrm{Standard}\;OD\;\mathrm{value}-\mathrm{Blank}\;OD\;\mathrm{value}\\ \times \mathrm{Concentration of standard}\\ ÷\mathrm{Protein}\;\mathrm{concentration}\;\mathrm{of}\;\mathrm{the}\;\mathrm{samples}\;to\;\mathrm{be}\;\mathrm{measured} \end{array}}$$

where the standards in the standard tubes are at a concentration of 10 nmol/mL, MDA is in nmol/mgprot, and the protein concentration of the samples to be tested is in mgprot/mL.

#### 2.5.3 Determination of superoxide dismutase activity

The T-SOD activity determination procedure followed the guidelines outlined in the T-SOD kit (using the WST-1 method). Subsequently, the T-SOD activity for each sample group was calculated using Equation 8 after the reaction was completed.


(8) $$\begin{array}{l} \mathrm{T-SOD viability}\\ =\frac{\mathrm{Control}\;OD\;\mathrm{value}-\mathrm{Measured}\;OD\;\mathrm{value}}{\begin{array}{l} \mathrm{Blank}\;OD\;\mathrm{value}\\ \times \frac{\mathrm{Total volume of reaction solution}\;\left(\mathrm{mL}\right)}{\mathrm{Sample size}\;\left(\mathrm{statistics}\right)\;\left(\mathrm{mL}\right)}÷\mathrm{protein content} \end{array}}÷50\% \end{array}$$

### 2.6 Investigation of *in vitro* anti-inflammatory activity of safflower fermentation solution

RAW264.7 cells were thawed from storage at −80°C, resuscitated, and the resulting cell suspension was inoculated into T25 cell culture flasks with 25 mL of fully cultivated DMEM medium. The cell culture was then incubated at 37°C with 5% CO_2_. When the cell fusion reached 80 to 90%, the RAW264.7 cell suspension in optimal condition was aspirated and mixed with 0.4% Trypan Blue solution in a 1:1 ratio. The resulting mixture was then placed on a cell counting plate, observed under a microscope, and the HaCaT cell suspension with a cell density of 1 × 10^5^ cells/mL was prepared for further experimentation. The safflower fermentation solution was diluted with basal DMEM medium to create concentrations of 40, 30, 20, 10, 5, 2.5, 1.25, and 0.625%.

#### 2.6.1 Evaluation of safe concentration of safflower fermentation solution on RAW264.7 cells

Sterile PBS solution, cell suspension, various concentrations of safflower fermentation solution, and basic DMEM medium were sequentially added to the 96-well cell culture plates. Subsequently, MTT solution was added and incubated for 20 h. After a 4-h incubation period, DMSO solution was added to measure the absorbance value, and the cell viability of HaCaT cells was calculated using Equation 2. For detailed experimental procedures and cell viability determination, please refer to section 2.3.1.

#### 2.6.2 Sample preparation of an anti-inflammatory cellular model of safflower fermentation solution

Lipopolysaccharide (LPS) is a highly immunoreactive molecule found in the outer membrane of bacteria. It has the ability to activate the body’s immune response and is commonly utilized to induce inflammation in cellular models (Ping et al., n.d.). In the control group, normal group, and model group, 100 μL of basal 1,640 medium was added. In the positive group, 100 μL of dexamethasone (100 μg/mL) was added. In the fermentation group, 100 μL of safflower fermentation solution of varying concentrations was added, respectively. The incubation was conducted for 4 h at 37°C under 5% CO_2_. After removing the 96-well plates following drug incubation, 1 μL of basal 1,640 medium was added to the normal group. For the remaining groups, 1 μL of LPS solution (100 μg/mL) was added to each well, resulting in a final LPS concentration of 1 μg/mL. Inflammatory stimulation was then conducted for 24 h at 37°C and 5% CO_2_ (Tongmee et al., 2021). After completion of stimulation by LPS, remove the 96-well plate, aspirate the supernatant from each well, centrifuge at 3,500 rpm for 15 min, and use the supernatant from each well as the sample for testing.

#### 2.6.3 Determination of TNF-α (tumor necrosis factor) in RAW264.7 cells

The standards in the TNF-α ELISA kit were diluted to concentrations of 0, 3.125, 6.25, 12.5, 25, 50, 100, and 200 pg/mL. Subsequently, the TNF-α standard curve was constructed following the guidelines provided in the TNF-α ELISA kit. The cell samples were processed following the guidelines of the TNF-α ELISA kit. The absorbance value of each sample was measured at 450 nm, and the TNF-α content in each well was determined based on the standard curve. Each group consisted of six parallel experimental groups.

#### 2.6.4 Determination of NO in RAW264.7 cells

A standard curve was plotted by diluting the standard NaNO2 to concentrations of 0, 1, 2, 5, 10, 20, 40, and 60 μM, following the instructions of the Nitric Oxide kit. Cell samples were then processed in accordance with the kit’s instructions, with the absorbance value of each sample measured at 540 nm. The NO content in each sample group was calculated using the established NO standard curve.

### 2.7 Investigation of *in vivo* anti-inflammatory activity of safflower fermentation solution

Zebrafish larvae with fluorescent neutrophil protein labeling were sorted into normal, model, positive, and various concentrations of safflower fermentation solution groups. Each group had three parallel experiments. The normal and model groups received 3 mL of zebrafish culture medium, the positive group received 3 mL of diluted dexamethasone (0.1 mg/mL) solution, and the fermentation solution group received 3 mL of safflower fermentation solution at different concentrations. Zebrafish larvae were incubated at 28°C for 20 h. Subsequently, a small amount of copper sulphate solution with a final concentration of 20 μM was added for a 2-h reaction to induce inflammation in the zebrafish larvae. Following this, the fish were rinsed with PBS and then anaesthetised with 0.1% tricaine. Neutrophil migration was observed individually under a fluorescence microscope, and the number of fluorescent neutrophils migrating was counted in each zebrafish to assess the anti-inflammatory activity of the safflower fermentation solution.

### 2.8 Safety evaluation of safflower fermentation solution

#### 2.8.1 Preparation of chick embryo chorionic allantoic membrane

In a fully self-incubating machine, chicken embryo eggs were incubated for 9 days. After removing the 9-day-old eggs, they were tested for egg illumination to mark the air chambers’ extent. Unfertilized, inactive, and deformed eggs were discarded. The chorionic allantoic membrane (CAM) preparation involved removing the eggshells above the air chambers using surgical forceps clamps. A 1 mL 0.9% NaCl solution was then applied along the edge of the air chamber membrane, completing the experiment within 20 min.

#### 2.8.2 Experimental method for eye irritation of safflower fermentation solution

The prepared CAM was carefully separated from the inner membrane along the shell wall using medical forceps clamps. The blood vessels of the chick embryo were then examined under an electron microscope to check for any signs of haemorrhage resulting from the procedure. If no haemorrhage was observed, further experiments were conducted. Two silicone rubber rings were subsequently attached to the blood vessels of chick embryos using medical forceps. One rubber ring was infused slowly with 0.1 mL of 0.9% NaCl solution, while the other received 0.1 mL of either the sample solution or the reference substance solution. The reaction was allowed to proceed for 3 min before being stopped. Subsequently, the fluid sample within the ring was washed with a small volume of 0.9% sodium chloride solution, allowing for the examination of blood vessels within the rubber ring of the chick embryo membrane under an electron microscope to assess for haemorrhage, haemolysis, and coagulation. Each chick embryo was assessed and scored based on the presence of haemorrhage, coagulation, and vascular melting as outlined in Equation 9. The irritating and corrosive properties of the experimental group were then evaluated according to Table 3.

**Table 3:** Results evaluation of endpoint scoring method.

Rating at the end of the line	Irritation classification
ES ≤ 12	Non-/lightly irritating/corrosive
12 < ES < 16	Moderately irritating/corrosive
ES ≥ 16	Severe irritant/corrosive


(9) $$ES=\frac{\begin{array}{l} \mathrm{Sum}\;\mathrm{of scores for haemorrhage},\mathrm{coagulation and vascular}\\ \mathrm{melting phenomena observed in}\;6\;\mathrm{chick embryos} \end{array}}{3}$$

### 2.9 Data analysis

The experimental results were analyzed using Prism GraphPad 10 software for data analysis and chart generation. All experimental data were averaged from three independent experiments and are expressed as mean ± standard deviation. Statistical analysis of the experimental results was conducted using a one-way ANOVA, with differences considered statistically significant at *p* < 0.05.

## 3 Results and discussion

### 3.1 Screening of dominant strains

**Figure 1:**
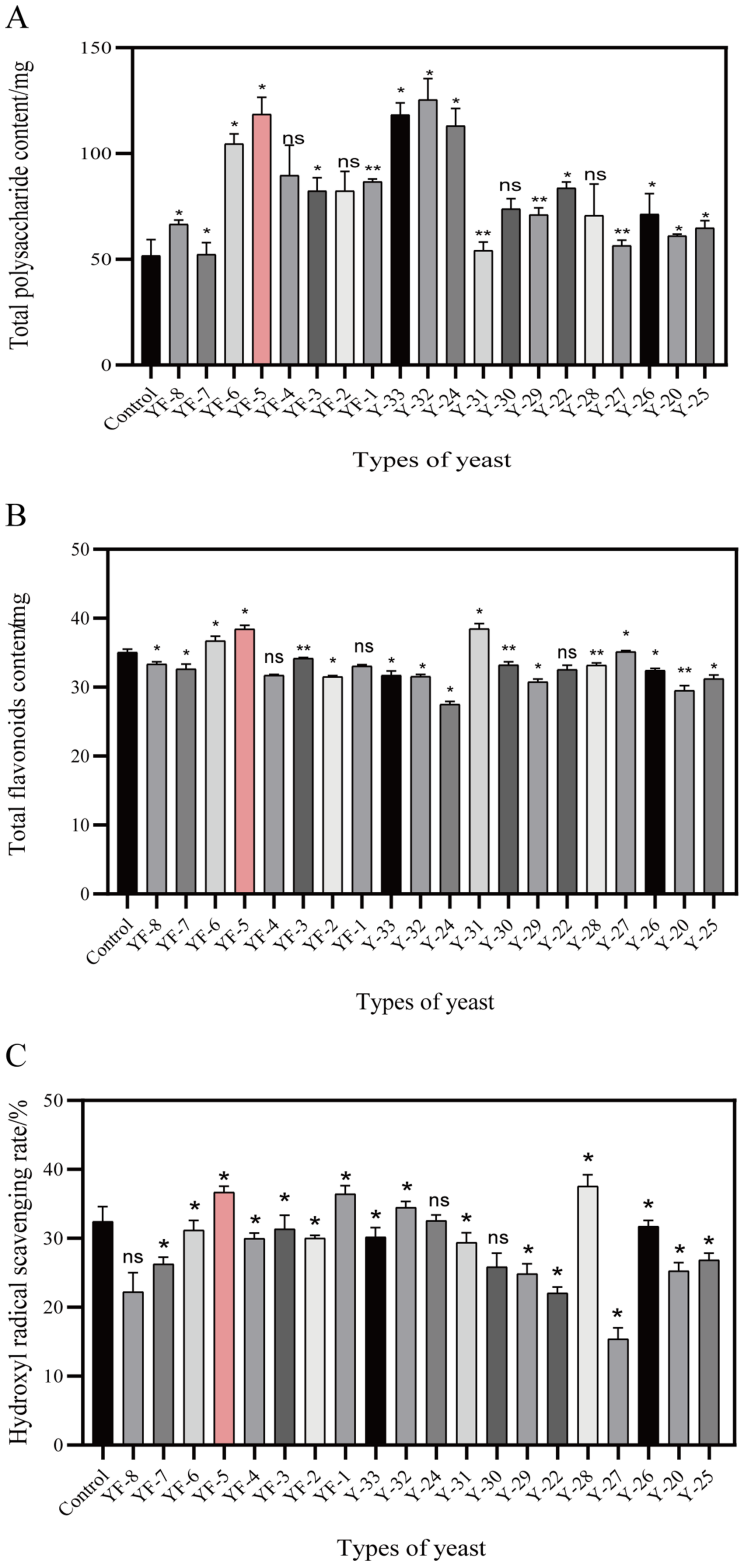
Screening of dominant strains. **(A)** The total polysaccharide content map of safflower fermented by different strains. **(B)** Total flavonoids content of safflower fermented by different strains. **(C)** Hydroxyl radical scavenging rate of each fermentation group. (Compared with the control group, ^*^p < 0.05).

In this study, 20 different yeast strains preserved in the laboratory were utilized to ferment safflower. The dominant strains for this fermentation process were selected based on their total polysaccharide content, total flavonoid levels, and the hydroxyl radical scavenging rate observed in the safflower fermentation solutions. The results, depicted in Figure 1, demonstrated that YF-5 exhibited a significant enhancing effect on these three indicators. A comparison between the safflower fermentation solution obtained from YF-5 fermented safflower and the control fermentation group revealed that the total polysaccharide content in the YF-5 safflower fermentation solution increased by 83.27% (Figure 1A), the total flavonoid content by 9.59% (Figure 1B), and the hydroxyl radical scavenging rate by 13.1% (Figure 1C). Consequently, YF-5 was identified as the most effective strain for safflower fermentation.

Yeast can utilize compounds found in plants for fermentation. Through its metabolic processes and transformations, yeast is capable of enhancing the concentration of existing bioactive compounds or synthesizing novel active substances (Xia et al., 2024).

### 3.2 Optimisation of fermentation conditions

#### 3.2.1 Single-factor experiment

Using the total flavonoid content in the safflower fermentation solution as an indicator, a single-factor method was employed to investigate the effects of various fermentation conditions—including fermentation temperature, bacterial inoculation amount, fermentation time, type of carbon source, carbon source concentration, and material-to-liquid ratio—on the total flavonoid content of safflower.

**Figure 2:**
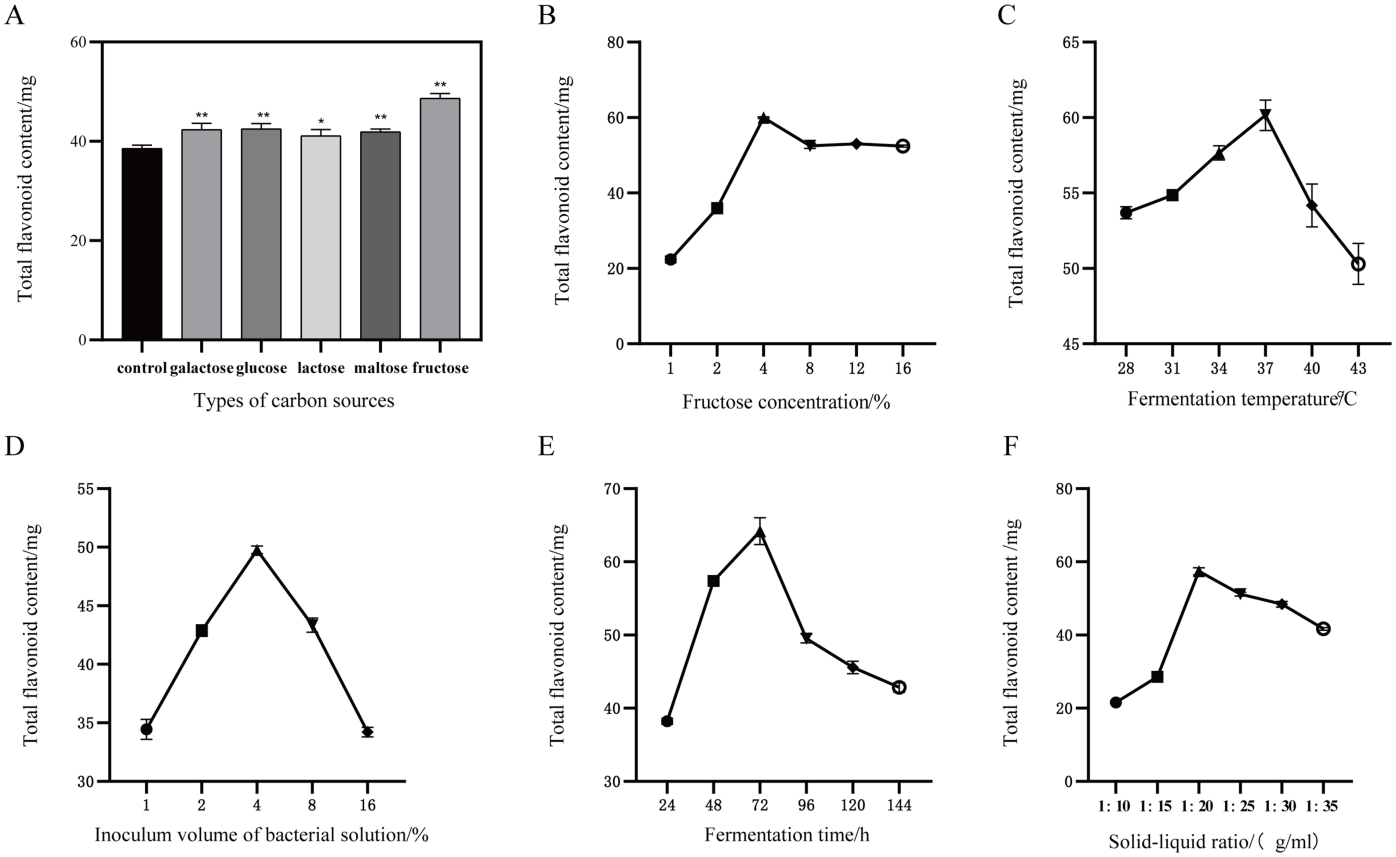
One-way experiment. **(A)** Types of carbon sources. **(B)** Fructose concentration. **(C)** Inoculum volume of bacterial solution. **(D)** Fermentation time. **(E)** Fermentation temperature. **(F)** Solid–liquid ratio. (Compared with the control group, ^*^p < 0.05 and ^**^p < 0.01).

Using the YF-5 fermentation group without an added carbon source as the control group, the results are presented in Figure 2A. All five carbon sources tested were found to enhance the total flavonoid content in the safflower fermentation solution, with fructose demonstrating the most significant effect. Compared to the control group, the total flavonoid content increased by 54.39%. Fructose was added to each fermentation group at concentrations of 1, 2, 4, 8, 12, and 16%, respectively. The results are presented in Figure 2B. Notably, when the fructose concentration reached 4%, the total flavonoid content in the fermentation solution was at its highest. At a fermentation temperature of 37°C, the total flavonoid content in the safflower fermentation solution reaches its peak, as illustrated in Figure 2C. When the bacterial inoculum amount was increased to 4%, the total flavonoid content in the safflower fermentation solution reached its maximum value. However, when the YF-5 bacterial inoculum amount exceeded 4%, the total flavonoid content in the safflower fermentation solution exhibited a downward trend (Figure 2D). After 72 h of fermentation, the total flavonoid content of YF-5 fermented safflower reached its maximum value (Figure 2E). When the material-to-liquid ratio was set at 1:20, the total flavonoid content in the safflower fermentation solution reached its maximum level (Figure 2F). However, as the ratio was increased beyond this point, the total flavonoid content gradually declined.

This experiment initially investigated the effects of fermentation temperature, bacterial inoculation amount, fermentation time, carbon source, carbon source concentration, and solid–liquid ratio on the total flavonoid content in safflower fermentation broth using a single-factor method. The results of the single-factor experiments are illustrated in Figure 2. The optimal values for each factor identified were: fructose as the preferred carbon source for YF-5 fermentation of safflower, a fructose concentration of 4%, a fermentation time of 72 h, a material-to-liquid ratio of 1:20, and a fermentation temperature of 37°C, with a bacterial inoculation amount of 4%.

Appropriate carbon sources can enhance yeast growth and optimize the quality of secondary metabolites. However, as the concentration of carbon sources increases further, the activity of yeast enzymes tends to decline, resulting in a decrease in fermentation efficiency (Gul et al., 2018). Fermentation temperature is a critical factor influencing the fermentation process. It significantly affects the growth of microbial strains, the activity of biological enzymes, and the progression of biochemical reactions (Du et al., 2022). The inoculation amount of fermentation bacteria liquid significantly influences the plant fermentation process. An insufficient inoculation amount may prevent the microorganisms from fully utilizing the safflower substrate, whereas an excessive inoculation amount may lead to the overutilization of the safflower substrate, resulting in premature termination of the fermentation. Fermentation time significantly influences the overall fermentation process, potentially altering the pharmacological effects of the fermentation broth and the concentration of active compounds (Tongmee et al., 2021). An appropriate fermentation duration allows microorganisms adequate time to complete the fermentation process, thereby enhancing the yield of effective substances.

#### 3.2.2 Results of the Plackett–Burman experiment

Design Expert 10 software was utilized to conduct the Plackett–Burman (PB) experiment, employing the total flavonoid content in the safflower fermentation solution as an indicator. This approach systematically analyzed the effects of fermentation temperature, bacterial inoculation amount, fermentation time, carbon source concentration, and material-to-liquid ratio on safflower fermentation. The study aimed to identify the factors that most significantly influence the total flavonoid content during the fermentation process.

A multivariate lumped model equation was developed using the results of the Plackett–Burman experiment to predict total flavonoid content (*R* = 51.47 + 3.77*A − 1.30*B + 18.08*C + 2.33*D + 4.50*E). The ANOVA analysis in Table 4 revealed a model *p*-value of 0.001, indicating its significance. The *R*^2^value of 0.9452 and corrected *R*^2^of 0.8995 suggest a good fit for the model. By examining the *p*-values of each fermentation condition, it was found that fructose concentration, feed-to-liquid ratio, and fermentation temperature had the most significant impact on the total flavonoid content of safflower.

**Table 4:** Plackett–Burman experimental analysis of variance table.

Factor	Sum of squares	Degrees of freedom	Mean square	*F*-value	*p*-value	Significance ranking
Model	4422.36	5	884.47	20.69	0.0010	Significant
A-Feed-to-liquid ratio	170.71	1	170.71	3.99	0.0927	—
B-Inoculum quantity	20.31	1	20.31	0.4749	0.5165	—
C-Fructose concentration	3923.65	1	3923.65	91.77	<0.0001	***
D-Duration of fermentation	65.13	1	65.13	1.52	0.2633	—
E-Fermentation temperature	242.57	1	242.57	5.67	0.0446	*
Residual	256.53	6	42.76			
Total	4678.89	11				

#### 3.2.3 Results of the Box–Behnken experiment

The Plackett–Burman (PB) experiment identified the three most significant factors influencing safflower fermentation and subsequently optimized the conditions for these factors using the Box–Behnken (BB) experimental design.

The detailed experimental results can be found in Table 5. According to the ANOVA in Table 6, it is concluded that the experimental model has a *p*-value <0.0001, indicating its significance. The analysis of the fitted model for the experimental model resulted in the model equation: *R* = 72.17 + 16.91*A + 1.50*B − 1.72*C + 1.04*AB − 1.48*AC + 2.07*BC − 21.55A2 −  8.76*B2 − 7.11*C2. Here, *R* represents the total flavonoid content of safflower. The model’s R-squared value is 0.9866, and the adjusted *R*-squared value is 0.9694, indicating a good fit for the model that can be utilized for predicting and analyzing safflower fermentation conditions (^***^p < 0.001).

**Table 5:** Box–Behnken test design and results table.

Group	A	B	C	Total flavonoid content (mg)
1	5	20	37	69.61
2	5	20	37	70.84
3	5	15	34	59.59
4	5	25	40	57.15
5	8	20	40	60.01
6	5	20	37	72.84
7	5	20	37	74.50
8	8	20	34	61.97
9	5	25	34	60.87
10	2	20	40	27.99
11	2	20	34	24.05
12	5	15	40	47.58
13	8	25	37	59.52
14	2	25	37	24.76
15	8	15	37	56.87
16	2	15	37	26.29
17	5	20	37	73.03

**Table 6:** Box–Behnken experimental analysis of variance table.

Factor	Sum of squares	Degrees of freedom	Mean square	*F*-value	*p*-value	Significance ranking
Model	5055.64	9	561.74	57.28	<0.0001	Significant
A-Fructose concentration	2287.98	1	2287.98	233.30	<0.0001	***
B-Feed-to-liquid ratio	17.92	1	17.92	1.83	0.2185	—
C-Fermentation temperature	23.69	1	23.69	2.42	0.1641	—
AB	4.36	1	4.36	0.4446	0.5263	—
AC	8.72	1	8.72	0.8887	0.3772	—
BC	17.16	1	17.16	1.75	0.2274	—
A^2^	1955.29	1	1955.29	199.38	<0.0001	***
B^2^	322.87	1	322.87	32.92	0.0007	***
C^2^	212.81	1	212.81	21.70	0.0023	***
Residual	68.65	7	9.81			
Lack of fit	53.72	3	17.91	4.80	0.0820	—
Pure error	14.93	4	3.73			
Cor. total	5124.28	16				

**Figure 3:**
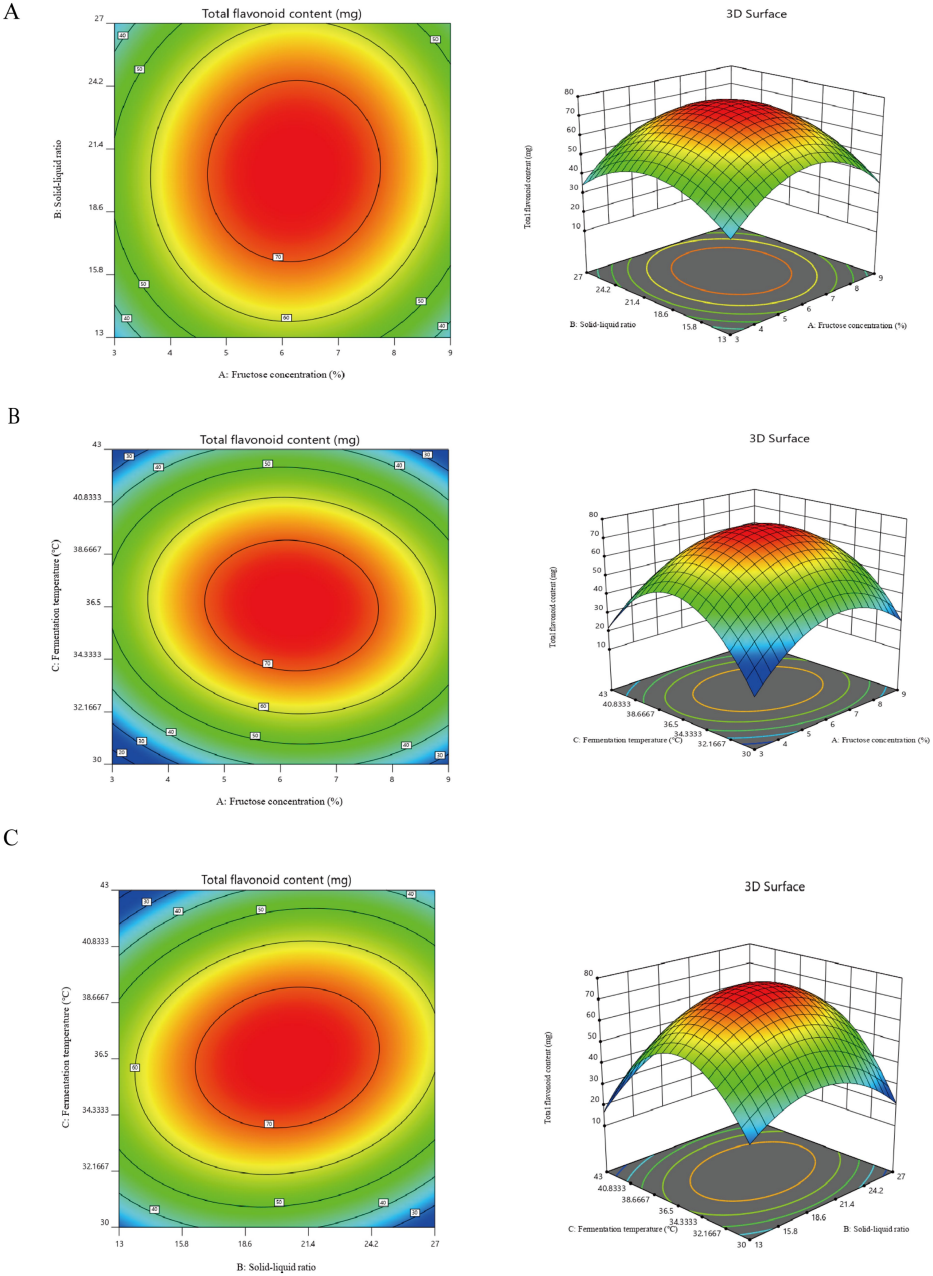
Results of the Plackett–Burman and Box–Behnken experiment. **(A)** Contours and response surface plots of the effects of fructose concentration and solid–liquid ratio on the total flavonoid content in safflower fermentation broth. **(B)** Contours and response surface plots of the effects of fructose concentration and fermentation temperature on the total flavonoid content in safflower fermentation broth. **(C)** The effects of solid–liquid ratio and fermentation temperature on the content of total flavonoids in safflower fermentation broth were analyzed by contour map and response surface map.

The response surface and contour plots of fermentation temperature, fructose concentration, and material-liquid ratio on the total flavonoid content of safflower were generated using the total flavonoid content in safflower fermentation solution as an indicator. These plots are depicted in Figures 3A–C.

The optimal values of the main factors were determined through software analysis to be a fermentation temperature of 36.55°C, a material-liquid ratio of 1:20.46, and a fructose concentration of 6.20%. Subsequently, the fermentation conditions were fine-tuned by adjusting decimal points. The ideal fermentation system for YF-5 fermentation of safflower was established with a fermentation temperature of 36.6°C, a material-liquid ratio of 1:20.5, a fructose concentration of 6.20%, a fermentation duration of 72 h, and a bacterial inoculum of 4%. Safflower fermentation experiments were conducted using this optimal fermentation system, with three parallel experiments performed. The results indicated that the actual total flavonoid content in the safflower fermentation broth, fermented under the optimal conditions, was 72.93 mg, which is in close proximity to the expected value of 75.45 mg.

The advantages of safflower fermentation solution as a novel cosmetic raw material stem from its natural and biodegradable properties. Additionally, the active ingredients produced during the fermentation process may enhance the efficacy of cosmetics. By optimizing the fermentation conditions, the total flavonoid content has been increased, thereby establishing a strong foundation for the application of safflower fermentation solution in the cosmetics industry.

### 3.3 Experiments on antioxidant activity of safflower fermentation solutions

#### 3.3.1 Effect of different concentrations of safflower fermentation solution on HaCaT cells

The MTT assay was employed to assess the effects of varying concentrations of saffron fermentation broth, along with 25 μg/mL of vitamin C (Vc) and hydrogen peroxide in the concentration range of 2 mM to 0.25 mM, on the viability of HaCaT cells. The appropriate concentration was subsequently selected for further experiments aimed at exploring the *in vitro* antioxidant activity.

**Figure 4:**
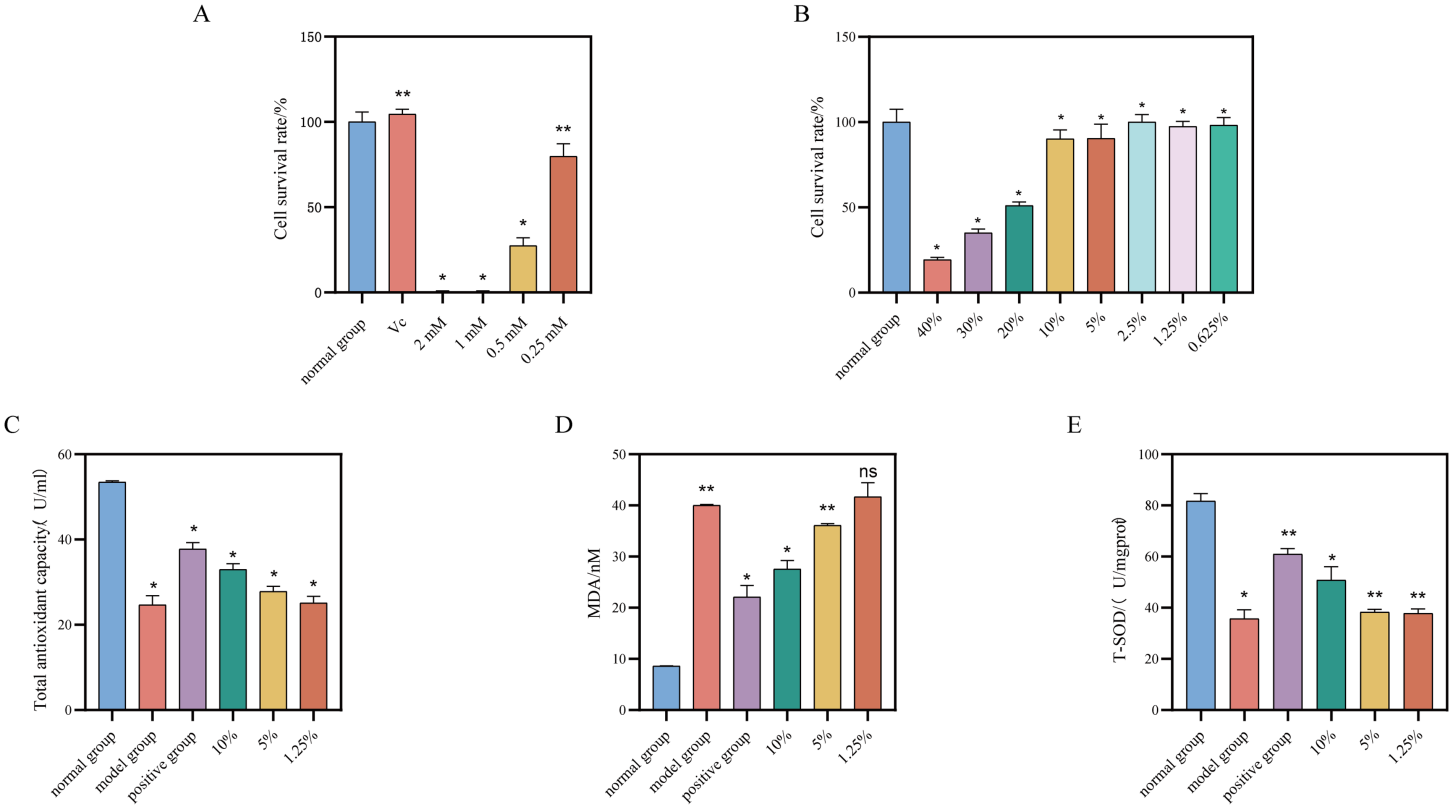
Effect of different concentrations of safflower fermentation solution on HaCaT cells. **(A)** The effect of different concentrations of hydrogen peroxide and vitamin C (25 μg/mL) on the viability of HaCaT cells. **(B)** The effect of different concentrations of safflower fermentation liquid on the viability of HaCaT cells. **(C)** Total antioxidant effects of different concentrations of safflower fermentation liquid on oxidatively damaged HaCaT cells. **(D)** The effect of different concentrations of safflower fermentation liquid on the MDA content in oxidatively damaged HaCaT cells. **(E)** The effect of different concentrations of safflower fermentation liquid on the SOD activity in oxidatively damaged HaCaT cells. (Compared to the normal group, ^*^p < 0.05 and ^**^p < 0.01).

The results of MTT experiments were computed using Equation 2 and depicted in Figure 4A. Vitamin C at a concentration of 25 μg/mL did not impact HaCaT cell activity. Hydrogen peroxide concentrations ranging from 2 mM to 0.5 mM inhibited HaCaT cell activity, while 0.25 mM had no effect. Subsequently, a model for HaCaT cell oxidative damage was established by stimulating the cells with hydrogen peroxide at a concentration of 0.25 mM, using 25 μg/mL of vitamin C as the positive control in subsequent antioxidant experiments.

The results from Figure 4B indicate that concentrations of safflower fermentation solution above 20% exhibited a notable inhibitory effect on HaCaT cells. However, this inhibitory effect gradually decreased as the concentration of safflower fermentation solution decreased below 20%, with HaCaT cell activity remaining above 50% within the concentration range of 10 to 0.625%.

Due to the high concentration of safflower fermentation solution, it may be toxic to cells, affecting cell growth and organelle function, resulting in reduced cell activity. Therefore, three concentrations of 10, 5, and 1.25% were chosen as high, medium, and low concentrations for the subsequent antioxidant activity evaluation experiments to investigate the antioxidant activity of safflower fermentation solution.

#### 3.3.2 Effect of safflower fermentation solution on total antioxidant capacity, malondialdehyde content and superoxide dismutase activity of HaCaT cells

The HaCaT cell oxidative damage model was treated with various concentrations of safflower fermentation solution. The total antioxidant capacity (T-AOC), malondialdehyde (MDA) content, and superoxide dismutase (SOD) activity of HaCaT cells were measured to assess the antioxidant capacity of the safflower fermentation solution.

The study examined how different concentrations of safflower fermentation solution affected the total antioxidant capacity of HaCaT cells, utilizing a T-AOC kit. Figure 4C displays the results, showing that low concentrations of safflower fermentation solution had a limited antioxidant capacity in the HaCaT cell oxidative damage model. In contrast, the 10% safflower fermentation solution exhibited a strong antioxidant effect similar to the positive control group.

The impact of different concentrations of safflower fermentation solution on MDA content in HaCaT cells was investigated utilizing an MDA kit. Figure 4D illustrates a decrease in MDA content in HaCaT cells with increasing safflower fermentation liquid concentration. Specifically, the MDA content of HaCaT cells treated with 10% safflower fermentation solution was measured at 27.57 nmol/mgprot, a level comparable to that of the positive control group. This finding provides evidence of antioxidant activity associated with safflower fermentation solution.

The impact of different concentrations of safflower fermentation liquid on superoxide dismutase (SOD) activity in HaCaT cells was assessed using the T-SOD kit. Figure 4E illustrates that the SOD activity of HaCaT cells treated with 10% safflower fermentation solution was 50.78 U/mgprot, comparable to the positive control group. Decreasing concentrations of safflower fermentation solution resulted in a gradual weakening of SOD activity in oxidatively damaged cells, suggesting that the safflower fermentation solution could mitigate the decline in SOD activity caused by oxidative stress in HaCaT cells, demonstrating certain antioxidant properties.

The phenolic components, Safflor Yellow A and Hydroxy Safflor Yellow A, exhibit high antioxidant activity and serve as the active constituents of safflower antioxidant. These compounds can diminish the susceptibility to lipid peroxidation and regulate oxidative stress in human skin fibroblasts (HuDe) at low concentrations (Bacchetti et al., 2020). Fermentation of safflower by yeast enhances the levels of its antioxidant active ingredients, thereby improving its antioxidant efficacy.

#### 3.3.3 Effect of safflower fermentation solution on the growth of embryos of zebrafish of the AB lineage

This study evaluated the effects of different concentrations of safflower fermentation solution on zebrafish embryos by measuring the hatching rate, survival rate, malformation rate, and other relevant indicators. Based on these evaluations, appropriate concentrations of safflower fermentation solution were selected for subsequent *in vivo* experiments exploring antioxidant activity.

**Figure 5:**
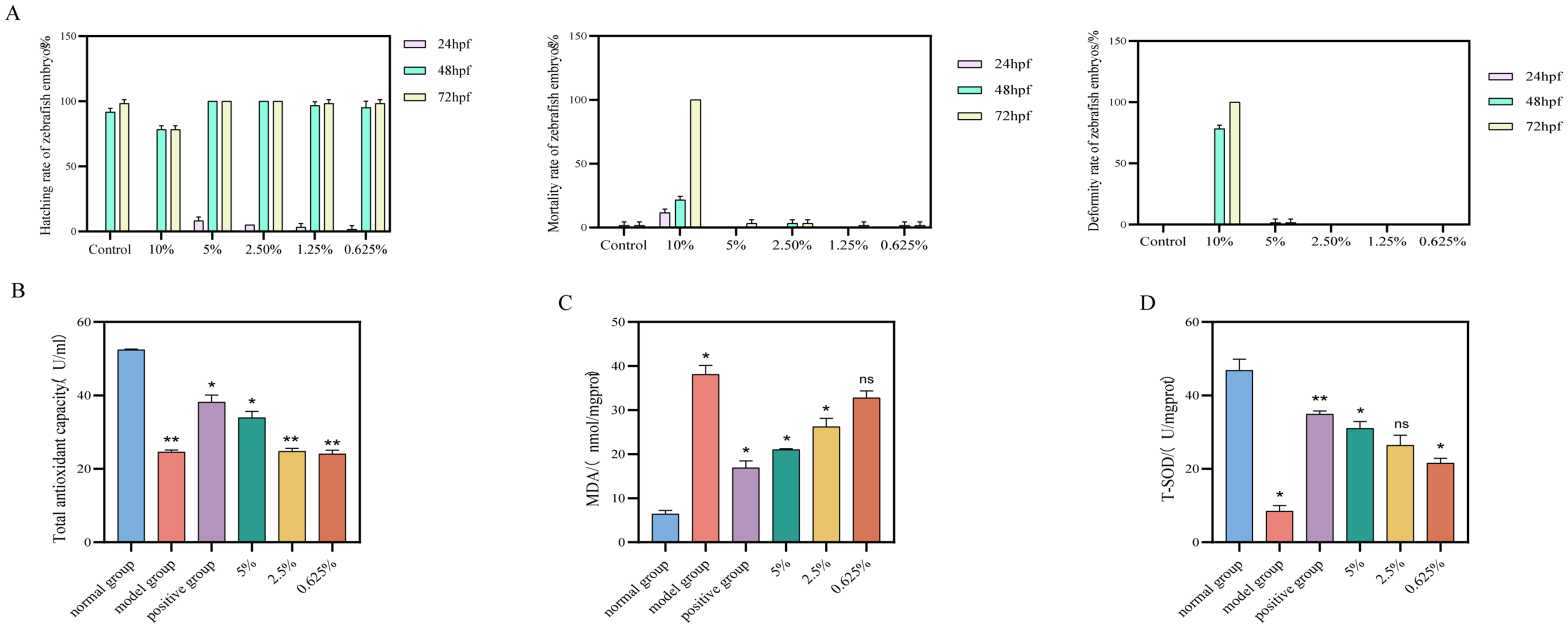
Effect of safflower fermentation solution on the growth of embryos of zebrafish of the AB lineage. **(A)** The effects of different concentrations of safflower fermentation broth on the hatching rate, mortality rate, and deformity rate of zebrafish embryos. Effect of safflower fermentation solution on total antioxidant capacity, MDA content, and SOD activity in an embryonic model of oxidative stress zebrafish. **(B)** The effect of different concentrations of safflower fermentation liquid on the total antioxidant capacity in zebrafish. **(C)** The effect of different concentrations of safflower fermentation liquid on the MDA content in zebrafish. **(D)** The effect of different concentrations of safflower fermentation liquid on the SOD activity in zebrafish. (Compared to the normal group, ^*^p < 0.05).

The concentrations of safflower fermentation solution tested on zebrafish embryos were chosen based on the hatching rate, mortality rate, and deformity rate of the embryos. The results, depicted in Figure 5A, revealed that a 10% concentration of safflower fermentation solution led to significant damage to the embryos, while higher concentrations proved to be toxic. Conversely, concentrations of 5% or lower were found to be non-toxic and even promoted hatching. As a result, concentrations of 5, 2.5, and 0.625% were selected as the high, medium, and low concentrations for the zebrafish experiment.

#### 3.3.4 Effect of safflower fermentation solution on total antioxidant capacity, MDA content, and SOD activity in an embryonic model of oxidative stress zebrafish

The antioxidant capacity of safflower fermentation solution was evaluated by measuring the total antioxidant capacity (T-AOC), malondialdehyde (MDA) content, and superoxide dismutase (SOD) activity in a zebrafish embryo model treated with varying concentrations of safflower fermentation solution.

The total antioxidant capacity of each group was determined using the T-AOC kit. The results, illustrated in Figures 5B, indicate that safflower fermentation solutions with concentrations below 2.5% exhibited low total antioxidant capacity, whereas a 5% safflower fermentation solution showed comparable total antioxidant capacity to the positive control group. This implies that the 5% safflower fermentation solution can mitigate the decline in total antioxidant capacity in zebrafish induced by oxidative stress from AAPH, demonstrating some antioxidant properties.

Malondialdehyde (MDA) content was assessed using an MDA kit, and calculations were performed for each group. The results can be seen in Figure 5C. In zebrafish, MDA content decreased as the concentration of safflower fermentation solution increased. Interestingly, the MDA content of zebrafish treated with 5% safflower fermentation solution was comparable to that of the positive control group. This suggests that the 5% safflower fermentation solution exhibits antioxidant activity in the zebrafish model of oxidative damage induced by AAPH stimulation.

The impact of varying concentrations of safflower fermentation solution on SOD activity in oxidatively damaged zebrafish was assessed using the T-SOD kit. Figure 5D illustrate that SOD activity in zebrafish increased proportionally with the concentration of safflower fermentation solution. This suggests that the safflower fermentation solution effectively inhibited the impact of AAPH on SOD within the zebrafish model of oxidative damage induced by AAPH stimulation. Furthermore, the SOD activities of the fermentation broth groups closely resembled those of the positive group, indicating that the safflower fermentation solution exhibits antioxidant properties.

As the concentration of safflower fermentation solution increases, the malondialdehyde (MDA) content in the zebrafish oxidative damage model gradually decreases, while superoxide dismutase (SOD) activity and total antioxidant capacity gradually increase. This indicates that safflower fermentation solution can inhibit oxidative damage to zebrafish induced by AAPH stimulation and possesses strong antioxidant capacity. In summary, safflower fermentation solution exhibits significant antioxidant activity and can serve as a novel cosmetic raw material with antioxidant properties, thereby providing a scientific basis for the development of antioxidant products.

### 3.4 Experimental anti-inflammatory activity of safflower fermentation solution

#### 3.4.1 Investigation of safe concentration of safflower fermentation solution against RAW264.7

The MTT experiment was conducted to assess the effects of varying concentrations of safflower fermentation solution and 1 μg/mL LPS on the activity of RAW264.7 cells. Appropriate concentrations were subsequently selected for further exploration of *in vitro* anti-inflammatory activity.

**Figure 6:**
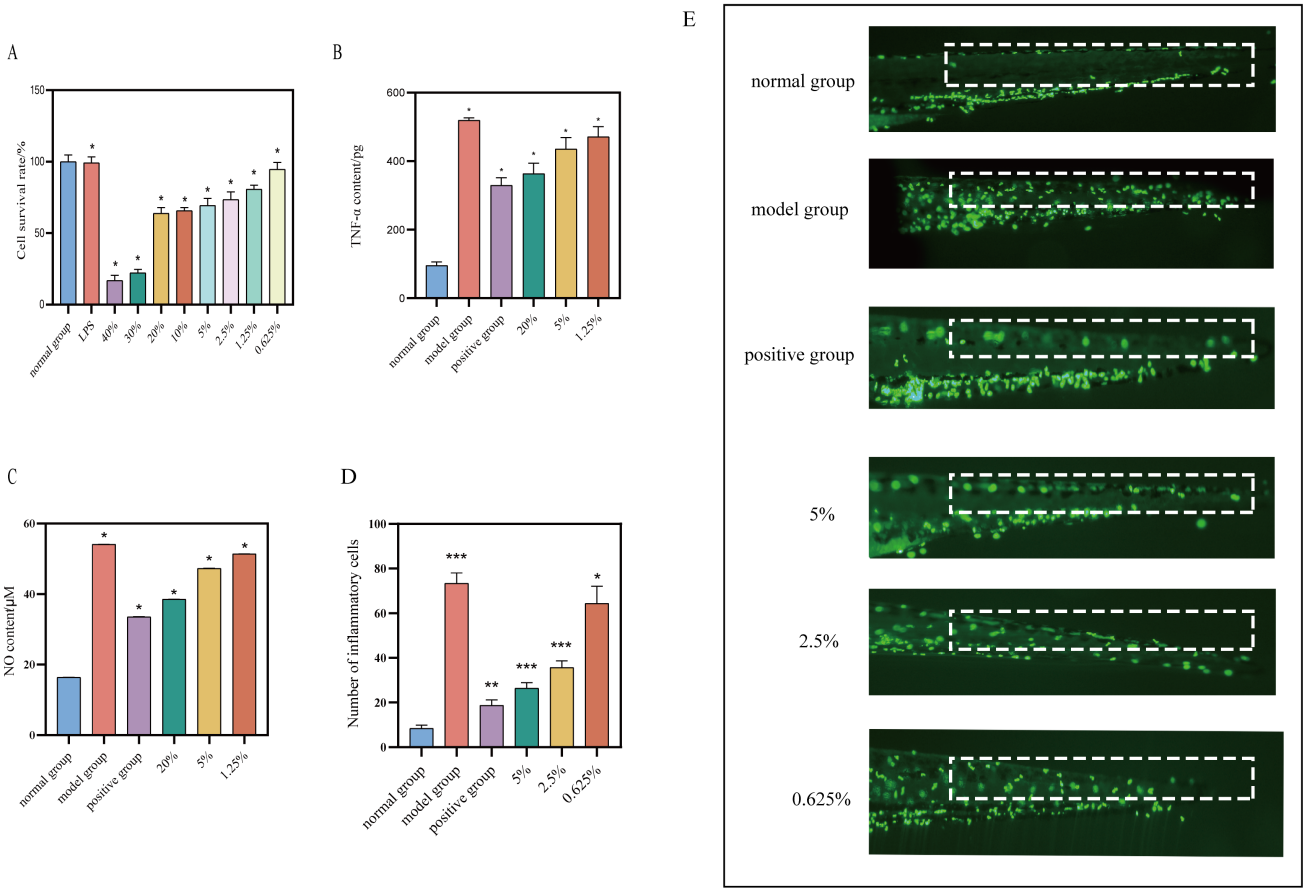
Investigation of safe concentration of safflower fermentation solution against RAW264.7. **(A)** The effect of different concentrations of safflower fermentation liquid on the viability of RAW264.7 cells. **(B)** The effect of different concentrations of safflower fermentation liquid on the TNF-α content. **(C)** The effect of different concentrations of safflower fermentation liquid on the NO content. (Compared to the normal group, ^*^p < 0.05). Effect of safflower fermentation solution on neutrophil migration in zebrafish *in vivo*. **(D)** The number of migrating fluorescent neutrophil proteins in zebrafish tails by different concentrations of safflower fermentation broths. **(E)** The effect of different concentrations of safflower fermentation broth on the migration of fluorescent neutrophils in the tail of zebrafish. (Compared to normal group, ^*^p < 0.05, ^**^p < 0.01, and ^***^p < 0.001).

The MTT assay was utilized to assess the impact of various concentrations of safflower fermentation solution and 1 μg/mL of LPS on RAW264.7 cells. The safflower fermentation solution was diluted into different concentrations using basal 1,640 medium. The results, illustrated in Figure 6A, indicated that LPS at a concentration of 1 μg/mL did not inhibit RAW264.7 cell activity. Notably, 30 and 40% safflower fermentation solutions exhibited a significant inhibitory effect on RAW264.7 cell activity, whereas concentrations below 20% had a milder impact, with the cell survival rate remaining above 50%. Consequently, LPS at 1 μg/mL was selected as the inflammation inducer, with safflower fermentation solutions at concentrations of 20, 5, and 1.25% chosen as high, medium, and low concentrations, respectively, for subsequent experiments investigating anti-inflammatory properties.

#### 3.4.2 Effect of safflower fermentation solution on TNF-α content in RAW264.7 inflammation model cells

The TNF-α standard curve was generated using the TNF-α ELISA kit’s instruction manual, with the equation *Y* = 193.5*X − 10.67 and a high *R*^2^ value of 0.9992, indicating a strong linear relationship. TNF-α levels were determined for each group as per the kit’s guidelines. Figure 6B shows that the TNF-α content in the model group was 5.44 times higher than in the normal group, with the positive group exhibiting a 63.34% inhibition rate on TNF-α content, confirming the successful establishment of the RAW264.7 cell inflammation model. The TNF-α levels in the safflower fermentation liquid samples decreased as the concentration increased. Specifically, when the concentration of safflower fermentation liquid was 20%, the TNF-α content was comparable to that of the positive group, suggesting that the 20% safflower fermentation liquid exhibited anti-inflammatory properties.

#### 3.4.3 Effect of safflower fermentation solution on NO content in RAW264.7 inflammation model cells

RAW264.7 cells were stimulated with 1 μg/mL of LPS to induce an inflammatory response, and the supernatant from each group of cells was gathered. A standard curve for NO was constructed following the guidelines provided in the NO kit manual, *Y* = 25.14*X − 4.845 and a high *R*^2^ value of 0.9982, indicating a strong linear correlation.

The collected cell supernatants were pre-treated and the absorbance values of each sample group were determined following the NO kit instructions. These values were then substituted into the NO standard curve to calculate the NO content of each group. The NO content of each group is depicted in Figure 6C. It was observed that the NO content decreases with increasing concentration of safflower fermentation solution. Interestingly, the NO content of the 20% concentration of fermentation broth was similar to that of the positive group, indicating that 20% of safflower fermentation solution exhibits strong anti-inflammatory activity.

#### 3.4.4 Effect of safflower fermentation solution on neutrophil migration in zebrafish *in vivo*

A zebrafish inflammation model was developed using copper sulfate to induce inflammation in zebrafish expressing fluorescent neutrophil proteins. By observing the migration of these proteins from the body surface to the tail of the zebrafish under a fluorescence microscope, the anti-inflammatory effects of the samples could be evaluated (Feng-ling et al., 2023). The results, depicted in Figures 6D,E, demonstrated a 64.09% reduction in the migration of fluorescent neutrophil proteins in the zebrafish treated with 5% safflower fermentation solution compared to the model group. This reduction was 0.7 times greater than that observed in the positive control group, indicating that the 5% safflower fermentation solution effectively attenuated the inflammatory response induced by copper sulfate in zebrafish.

The compound (5R)-5-acetoxy-8,10,12-tetradecanoyl-1-O-β-D-glucopyranoside, isolated from safflower, significantly inhibits nitric oxide (NO) production in mouse monocyte macrophage leukemia cells (RAW264.7), thereby exerting anti-inflammatory effects (Li et al., 2021). The fermentation process enhances the concentration of anti-inflammatory active ingredients in safflower fermentation solution, thereby improving its anti-inflammatory efficacy.

## 4 Safety evaluation of safflower fermentation solution

The safety of the safflower fermentation solution was assessed using the eye-irritating chick embryo chorionic allantoic membrane (CAM) test, thereby establishing a scientific foundation for the safe incorporation of this ingredient in cosmetic products.

**Figure 7:**
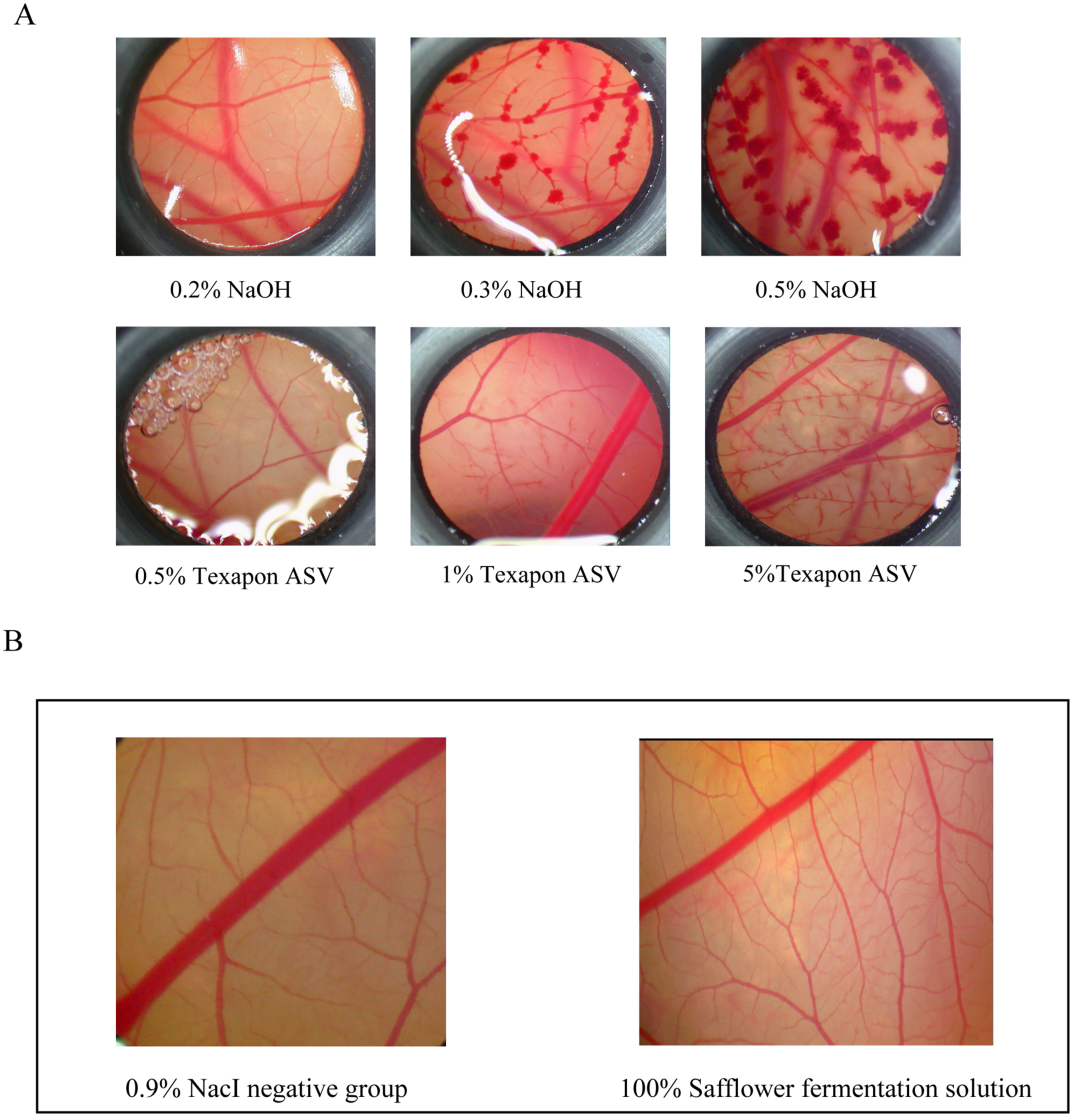
Safety evaluation of safflower fermentation solution. **(A)** Different concentrations of the reference substance control CAM diagram. **(B)** CAM diagram of 100% safflower fermentation broth.

A 0.9% sodium chloride solution was utilized as the solvent control, while the fatty alcohol sodium salt of sulphate (Texapon ASV) and sodium hydroxide served as baseline controls. ASV at concentrations of 0.5, 1.0, and 5% was applied to the CAM for 3 min, and the resulting effects on the blood vessels within the circle were observed. Similarly, NaCl at concentrations of 0.2, 0.3, and 0.5% was applied to the CAM for 3 min, and the effects on the blood vessels were also observed. Baseline substance control charts, categorized by severity as mild coagulation, moderate coagulation, and severe coagulation, were used to assess the toxicity of the samples on the CAMs. The impact of each concentration of the baseline substance on the CAM vasculature is depicted in Figure 7A.

CAMs were stimulated with safflower fermentation solution at concentrations of 100 and 0.9% NaCl for 3 min. The results, depicted in Figure 7B, revealed that the 100% safflower fermentation solution did not induce significant damage to the CAM blood vessels. There was no evidence of vascular hemorrhage, coagulation, or melting, and the ES score was 0. These findings suggest that the safflower fermentation solution was non-irritating to the CAM blood vessels, thus providing a foundational safety assessment for its potential use as a novel cosmetic ingredient in the beauty care industry.

## 5 Conclusion

Microbial fermentation technology was employed to produce safflower fermentation solution, and its antioxidant, anti-inflammatory, and other biological activities were investigated for use as a novel natural cosmetic raw material in cosmetic formulations. In this study, YF-5 was identified as the dominant strain for safflower fermentation from a selection of 20 yeast strains. The fermentation conditions were optimized using a single-factor experiment and response surface methodology. Following the optimization, the total flavonoid content in the safflower fermentation solution increased by 1.56 times compared to the levels prior to optimization and by 1.87 times compared to the blank fermentation group. This study utilized modern microbial fermentation technology; however, it encountered challenges during the fermentation process, including variations in fermentation strains and the inability of strains and key enzyme activities to maintain optimal levels throughout the fermentation. With the advancements in genetic engineering and synthetic biology, technologies related to synthetic biology can be employed to construct ‘cell factories’ that efficiently synthesize specific types of active substances for incorporation into cosmetic raw materials.

The study of the antioxidant and anti-inflammatory activities of safflower fermentation solution revealed that it can mitigate oxidative damage induced by hydrogen peroxide and AAPH, demonstrating notable antioxidant activity both *in vivo* and *in vitro*. Furthermore, safflower fermentation solution reduces the inflammatory response in RAW264.7 cells stimulated by LPS and also attenuates the inflammatory response in zebrafish induced by copper sulfate, indicating its significant anti-inflammatory activity in both *in vivo* and *in vitro* settings. The antioxidant and anti-inflammatory properties of safflower fermentation solution may be attributed to the active compounds present in safflower. Microbial fermentation can enhance and concentrate these active substances. However, the specific mechanisms underlying the anti-inflammatory and antioxidant effects of safflower fermentation solution remain unclear, warranting further investigation and exploration in future studies.

This study evaluated the safety of safflower fermentation solution using the chick embryo chorioallantoic membrane assay. For the subsequent development of products containing safflower fermentation solution, it is essential to conduct additional types of acute toxicity experiments on animals and utilize 3D skin models to ensure a more comprehensive safety assessment, thereby facilitating the effective application of safflower fermentation solution in the cosmetic industry.

## Data Availability

The original contributions presented in the study are included in the article/supplementary material, further inquiries can be directed to the corresponding authors.
